# *JPGN Reports*: 2024 and beyond

**DOI:** 10.1002/jpr3.12030

**Published:** 2023-12-27

**Authors:** Dominique Belli, Sandeep K. Gupta, Renata Auricchio

**Affiliations:** ^1^ Welcome Center HUG‐Unige‐HeSSo University of Geneva Geneva Switzerland; ^2^ Division of Pediatric Gastroenterology, Hepatology, and Nutrition, Department of Pediatrics University of Alabama at Birmingham Birmingham Alabama USA; ^3^ Department of Translation Medical Science, Pediatric Section, European Laboratory for the Investigation of Food Induced Disease (ELFID) University Federico II Naples Italy

We want to wish everyone a Happy New Year; this year, 2024, is a significant milestone in the life of our journal, *JPGN Reports*. Not only does it mark our 4th anniversary but also a change in our publisher from Wolters Kluwer to Wiley. It has been a fantastic journey of growth and collaboration with Wolters Kluwer for the last few years, and for which we are grateful to the Wolters Kluwer team. Under the guidance of our parent societies, ESPGHAN and NASPGHAN, we the Editors‐in‐Chief along with our editorial teams are excited to work with Wiley and look forward to a bright and exciting future over the coming years.

With this transition we have been intentional and mindful of limiting the changes you would notice. For our authors and reviewers, the processes, including the use of the Editorial Manager system remain similar to the publisher change. The reader will note some differences such as page layout; however, *JPGN Reports* will continue to be an online‐only, open‐access publication, and the quality of content will continue, as before, to be excellent. Our teams will continue the many initiatives we have introduced over the last few years including the *Fellow/Young Reviewer* and the *Fellow Editor* Programs, and collaborations with external partners.

Again, we thank our Wolters Kluwer colleagues, Ali Manieri and Marianna Hagan, for their services, collegiality, and collaborative spirits. We welcome Brian Coughlin, Laura Bolte, and other team members from Wiley. We are grateful to Managing Editor Phyllis Barr for her assistance including during this publisher transition period.

We aim to continue engaging our authors, members, and readers, while elevating the scientific content, citations, and impact, as well as the visual aspects of the journal. As we strive to make JPGN Reports a destination of choice, we are excited about the bright future with Wiley. Above all, we thank you for being an integral part of our journey.

## CONFLICT OF INTEREST STATEMENT

The authors declare no conflict of interest.

